# Immobilization of single argon atoms in nano-cages of two-dimensional zeolite model systems

**DOI:** 10.1038/ncomms16118

**Published:** 2017-07-17

**Authors:** Jian-Qiang Zhong, Mengen Wang, Nusnin Akter, John D. Kestell, Alejandro M. Boscoboinik, Taejin Kim, Dario J. Stacchiola, Deyu Lu, J. Anibal Boscoboinik

**Affiliations:** 1 Center for Functional Nanomaterials, Brookhaven National Laboratory, Upton, New York 11973, USA; 2 Department of Materials Science and Chemical Engineering, Stony Book University, Stony Brook, New York 11790, USA; 3 Instituto de Fisica Aplicada INFAP-CONICET-Departamento de Fìsica-Universidad Nacional de San Luis, Chacabuco 917-5700-San Luis, Argentina

## Abstract

The confinement of noble gases on nanostructured surfaces, in contrast to bulk materials, at non-cryogenic temperatures represents a formidable challenge. In this work, individual Ar atoms are trapped at 300 K in nano-cages consisting of (alumino)silicate hexagonal prisms forming a two-dimensional array on a planar surface. The trapping of Ar atoms is detected *in situ* using synchrotron-based ambient pressure X-ray photoelectron spectroscopy. The atoms remain in the cages upon heating to 400 K. The trapping and release of Ar is studied combining surface science methods and density functional theory calculations. While the frameworks stay intact with the inclusion of Ar atoms, the permeability of gasses (for example, CO) through them is significantly affected, making these structures also interesting candidates for tunable atomic and molecular sieves. These findings enable the study of individually confined noble gas atoms using surface science methods, opening up new opportunities for fundamental research.

## Abstract (web summary)

While noble gases can be trapped in 3D porous structures, immobilizing them on 2D surfaces represents a formidable challenge. Here, the authors cage individual argon atoms in 2D model zeolite frameworks at room temperature, providing exciting opportunities for the fundamental study of isolated noble gas atoms using surface science methods.

Immobilizing single atoms of noble gases at room temperature within a two-dimensional (2D) array of nano-sized cages is both fundamentally interesting and technologically relevant. This could allow the vast artillery of surface science tools to study with exquisite level of detail the behaviour of individual unreactive atoms and molecules in nano-confinement, as well as the mechanism by which they enter and exit the cages. In addition, the knowledge gained using this approach could provide guidelines for the design, selection and improvement of adsorbent materials and membranes for gas separation^1^,^2^ and reprocessing of used nuclear fuel^3^,^4^.

Excellent candidates for these cages are found in 2D (alumino)silicate bilayer frameworks, consisting of an array of polygonal prisms forming a 2D self-containing structure, that interacts weakly with a single crystal metal substrate onto which is grown^5^,^6^,^7^. These frameworks have been proposed as surface science zeolite models given their similar chemical behaviour^8^. In contrast to other 2D materials such as graphene with only one atomic carbon layer, the cages in the 2D zeolite model systems allow the possibility of hosting atoms and molecules within the structure. The passage of single Pd and Au atoms through the cages onto the interface with the underlying metal substrate has been previously explored^9^,^10^. While previous work on these novel 2D materials focused on their study as catalysts^11^, their study for non-cryogenic trapping and separation of gases is also of critical importance^12^. An industrially relevant application is the separation of unreactive volatile species such as noble gases. Trapping noble gases on surfaces and especially within nano-sized cages similar to those of zeolites can provide an unprecedented level of detail on the adsorption processes when surface science tools are used, which may further stimulate the manipulation of these trapped gases with atomic precision. Note that trapping of noble gases has been previously achieved using three-dimensional porous materials^13^,^14^,^15^. Surface immobilization of noble gases can be typically achieved by condensation at cryogenic temperatures^16^. However, immobilizing noble gases on a surface at room temperature and above is challenging, and to our knowledge it has only been achieved by ion implantation^17^ by accelerating ions onto a surface. Electrostatic trapping of Xe has been reported but still at cryogenic temperatures^18^.

Aside from the cages within the framework, a second type of confined spaces is located between the 2D framework system and the metal support. This also allows the size selective diffusion of small molecules to intercalate at this interface^19^,^20^,^21^,^22^. Furthermore, the intercalation of molecules at the interface pushes the weakly bound 2D framework further away from the metal support. Previous reports show that the 2D silica film is permeable to small molecules, such as H_2_, CO and O_2_ (refs 19, 20, 21, 22), with a diffusion energy barrier of 0.5 eV for CO to go through the hexagonal prisms recently calculated from density functional theory (DFT)^21^. Note here the analogy with 3D zeolites, readily used as adsorbents and molecular sieves^13^,^23^. Another noble gas, xenon, has been shown to adsorb on zeolite chabazite, which is composed of the same secondary building block (the hexagonal prism) that makes up the 2D (alumino)silicate framework used here^24^.

Here, we show that the aforementioned 2D-zeolite models can trap individual Ar atoms in the nano-cages that make the surface, providing a new playground for the fundamental study of isolated noble gas atoms in confinement with surface science methods. This is demonstrated by ambient pressure X-ray photoelectron spectroscopy and by infrared reflection absorption spectroscopy. Comparison of the experimental data with DFT calculations provides more detailed fundamental insights, as discussed below.

## Results

### Trapping argon in nano-cages

**Figure 1:**
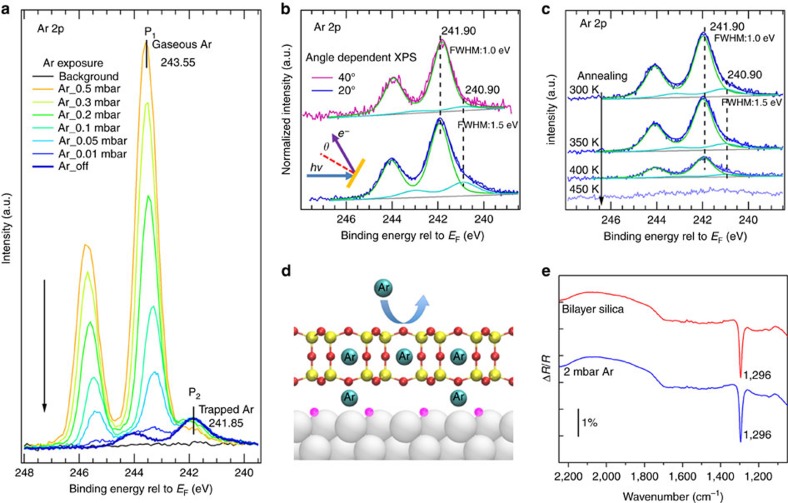
Ar trapping in the silica bilayer. XPS core level spectra of (**a**) pressure dependent Ar 2p, (**b**) angle dependent Ar 2p, (**c**) temperature dependent Ar 2p. (**d**) Schematic illustration of the trapped Ar atoms in the bilayer silica/Ru(0001). Colour code: Si (yellow), O in silica films (red), O chemisorbed on Ru(0001) (pink), Ru (silver) and Ar (cyan). (**e**) IRRA spectra of as-prepared silica bilayer and Ar-containing silica bilayer. The black spectrum in **a** is obtained in UHV prior to the Ar exposure, while the dark blue spectra in (**a**) and (**b**) are obtained in UHV after the Ar exposure. (Photon energy, *hv*=1000, eV; photoelectron emission angle, *θ*=20°).

Well-ordered 2D silica (SiO_2_) and aluminosilicate (Al_0.16_Si_0.84_O_2_) frameworks were grown on a Ruthenium(0001) surface, as described in detail elsewhere^5^,^25^. Trapping of Ar atoms in the frameworks is demonstrated *in situ* by Ambient Pressure X-ray Photoelectron Spectroscopy (AP-XPS). The 2D framework is first exposed to 0.5 mbar Ar. The AP-XPS spectrum in Fig. 1a shows a strong Ar 2p_3/2_ peak at 243.55 eV (P_1_) and a weak Ar 2p_3/2_ peak at 241.85 eV (P_2_). As the Ar pressure is lowered, the P_1_ peak gradually decreases and eventually disappears, while the P_2_ peak still remains even after evacuating the gas. P_1_ can then be assigned to gas phase Ar. Reference experiments carried out on a clean Ru(0001) surface show the gas phase Ar peaks only (Supplementary Fig. 1) and no evidence of Ar on the surface after evacuation, indicating conclusively that P_2_ in Fig. 1a corresponds to Ar trapped by the silica framework. It should be noted that Ar atoms are trapped only when the silica film is exposed to modest Ar pressures (>0.2 mbar) during the XPS experiments. However, for infrared reflection absorption spectroscopy (IRRAS) experiments described later, higher pressures (>2 mbar) are needed to trap Ar, indicating that the X-ray beam plays a role in facilitating the capturing of the atoms. This is very likely related to the ionization of Ar atoms by X-rays as part of the measurements. Careful analysis of P_2_ after evacuation (dark blue in Fig. 1a,b) shows that this peak has a main component at 241.90 eV and a shoulder at 240.90 eV. Angle dependent XPS experiments were carried out to qualitatively determine the relative depth at which these Ar atoms are located. As shown in Fig. 1b, the shoulder at lower binding energies becomes comparatively weaker at the larger *θ*=40° photoelectron emission angle (with respect to the surface normal) when compared to spectra taken at *θ*=20°. This indicates that the shoulder corresponds to Ar atoms at a deeper location and are assigned to the interface between the silica framework and the Ru(0001) surface (Ar_inter_) (Fig. 1d), while the main peak is assigned to Ar atoms within the hexagonal prism cages (Ar_cage_). The peak assignment is confirmed by DFT calculations at *Θ*=0.25 (*Θ* is the total coverage of trapped Ar per nano-cage), where the Ar 2p_3/2_ binding energy (*E*_BE_) of Ar_cage_ is 1.12 eV higher than that of the Ar_inter_ (see Supplementary Table 1 for details).

By comparing the ratio of peak areas between the Ar 2p and Si 2p (Supplementary Fig. 2), the total coverage of trapped Ar atoms was estimated at *Θ*=0.15. Note that the potential existence of larger polygonal prisms, in addition to the hexagonal prisms cages, may result in underestimating the coverage as the total density of hexagonal prism cages may be smaller^26^. The amount of trapped Ar at the Silica/Ru(0001) interface can be controlled by the distance between the framework and Ru(0001), which is determined by the coverage of chemisorbed oxygen on Ru(0001). This distance ranges between 3.85 Å (silica/(2 × 2)−3O/Ru(0001)) and 2.75 Å (silica/Ru(0001))^27^,^28^. Supplementary Fig. 3 shows that there is much less Ar trapped at the interface for the silica bilayer with less chemisorbed oxygen due to the smaller distance between the silica bilayer and the Ru(0001) surface^27^,^28^.

Upon annealing the sample to 400 K (Fig. 1c), the area of the Ar 2p peak decreases and the Ar_inter_/Ar_cage_ ratio is reduced from 0.24 to 0.11, suggesting that the Ar atoms trapped at the interface are less stable than those in the nano-cages. It is worth noting that the inclusion of Ar atoms in silica bilayer films does not change the characteristic phonon vibration frequency of the framework associated with the Si–O–Si linkage perpendicular to the surface plane at 1,296 cm^−1^, as shown in IRRA spectra in Fig. 1e. This is discussed further later in relation to lattice distortions obtained from DFT calculations.

**Figure 2:**
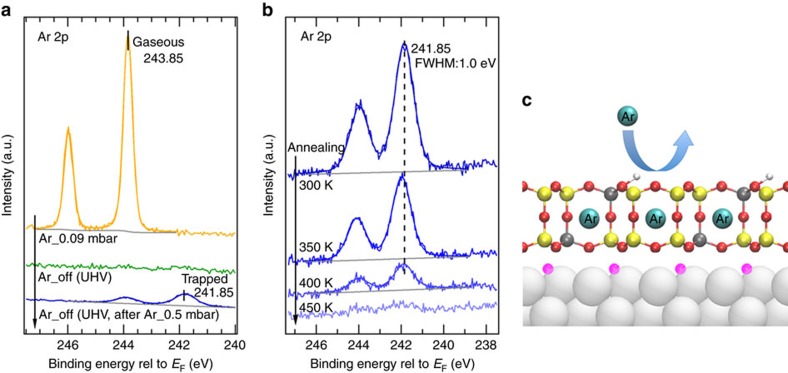
Ar trapping in the bilayer aluminosilicate (Al_0.16_Si_0.84_O_2_). (**a**) XPS core level spectra of Ar 2p taken under 0.09 mbar Ar (orange spectrum), under UHV after 0.09 mbar Ar exposure for 10 min (green spectrum), and under UHV after 0.5 mbar Ar exposure for 10 min (dark blue spectrum). (**b**) Temperature dependent UHV XPS core level spectra of Ar 2p. (**c**) Schematic illustration of the trapped Ar atoms in the bilayer aluminosilicate (Al_0.16_Si_0.84_O_2_). Colour code: Si (yellow), Al (dark grey), O in silica films (red), O chemisorbed on Ru(0001) (pink), H (light grey), Ru (silver) and Ar (cyan). (Photon energy, (**a**) *hv*=330 eV and (**b**) *hv*=1000, eV; photoelectron emission angle, *θ*=20°).

The trapping of Ar is also investigated for aluminosilicate bilayer frameworks, in which 16% of the Si atoms are substituted by Al (Al_0.16_Si_0.84_O_2_) (ref. 5). The top spectrum in Fig. 2a was taken at 0.09 mbar of Ar, where gas-phase Ar is clearly observed. No evidence of Ar is seen in the spectrum taken after evacuation (green spectrum in Fig. 2a). The surface was then exposed to 0.5 mbar of Ar and the blue spectrum in Fig. 2a was taken after evacuation, clearly showing that now Ar remains on the surface. A closer inspection reveals that there is only one type of Ar trapped (dark blue spectra in Fig. 2a,b), corresponding to atoms trapped in the cages. For the aluminosilciate bilayer, it is known that Al populates the bottom layer of the bilayer framework first for low Al contents (*x*<0.25) (ref. 5). Since the incorporation of Al in the framework comes along with the formation of negative charges (in zeolites these are compensated by cations), it is suggested that the Ru substrate may provide the charge compensation, explaining the preferential location of Al in the framework layer closest to the Ru(0001) surface. This results in additional electrostatic forces reducing the distance between the bilayer film and the Ru(0001) substrate, leaving no space for Ar atoms in this interfacial confined space, as depicted in Fig. 2c. As it was the case for silica, the trapped Ar atoms completely desorb only upon heating to 450 K, as shown in Fig. 2b. The presence of Ar atoms in these silica bilayer films does not change the electronic properties of the films (Supplementary Figs 4 and 5), as evident by the lack of change in binding energies of the Si 2p, O 1s, Al 2s and Ru 3d core levels.

**Figure 3:**
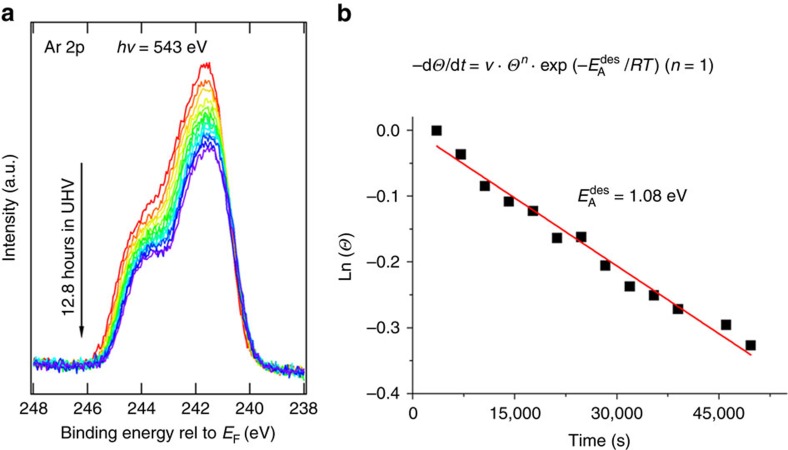
Kinetics of Ar desorption. (**a**) Time dependent XPS core level spectra of Ar 2p for the Ar trapped bilayer aluminosilicate (Al_0.2_Si_0.8_O_2_). (**b**) Plot of the measured Ar peak areas using the Polanyi–Wigner equation.

To further examine the kinetics of desorption of Ar atoms from the nano-cages, time dependent XPS was carried out for an aluminosilicate (Al_0.2_Si_0.8_O_2_) bilayer film at room temperature. The spectra as a function of time are shown in Fig. 3a and the peak area as a function of time is shown in Fig. 3b, from which the rate of desorption, **r**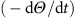, can be obtained. According to the Polanyi–Wigner analysis^29^, the desorption rate follows an Arrhenius-type behaviour


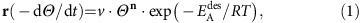


where *v* is the frequency factor, **n** is the kinetic order of desorption and 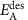 is the activation energy of desorption. For simple molecular desorption, a first-order process can be assumed with a frequency factor of ∼10^13^^±^^3^ s^−1^. The calculated activation energy for Ar desorption is ∼104.1±17.2 kJ mol^−1^ (∼1.08±0.18 eV). It should be noted that the exponential pre-factor and desorption energy may depend on the coverage of trapped Ar even for this simple system. The frequency factor used in this assumption is the one commonly chosen, when using the Redhead equation^30^ to estimate activation energies from temperature programmed desorption experiments. Our experimentally derived activation energy is in good agreement with the DFT calculations, as discussed below. Note however that a temperature dependent study would lead to a more accurate estimate of the activation energy, since in that case the assumption of the frequency factor would be eliminated.

### DFT calculations

**Figure 4:**
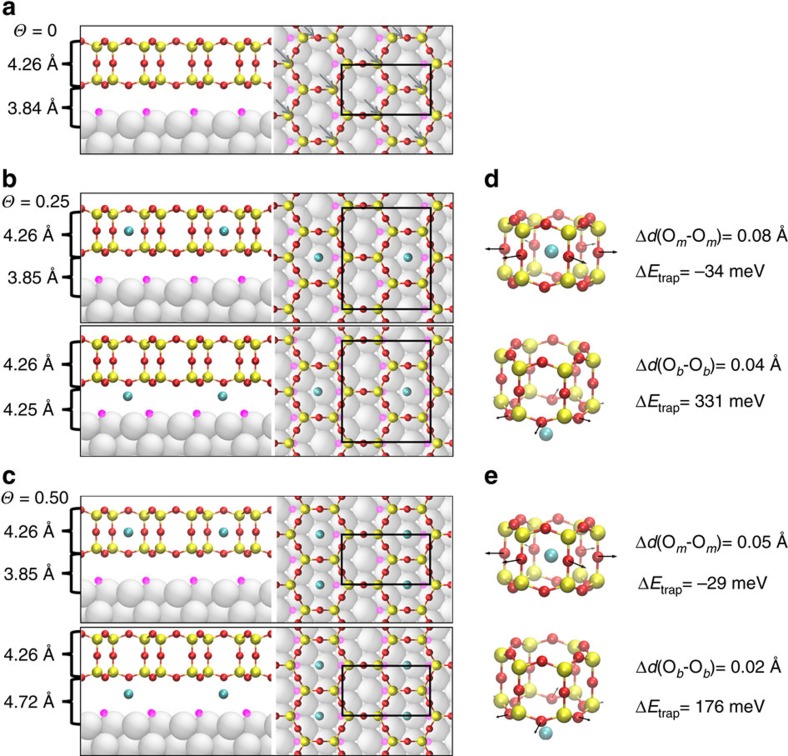
Geometric structures and calculated trapping energies. Side (left) and top (right) views of relaxed structures of (**a**) silica bilayer before Ar trapping ((SiO_2_)_8_/4O/Ru(0001)), (**b**) Ar-(SiO_2_)_16_/8O/Ru(0001) with an Ar coverage of 0.25, where Ar is trapped in the cages (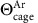
=0.25) or at the interface (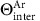
=0.25) and (**c**) Ar-(SiO_2_)_8_/4O/Ru(0001) with an Ar coverage of 0.50, where Ar is trapped in the cages (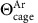
=0.50) or at the interface (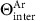
=0.50). Black rectangles represent the unit cell. Distances on the left correspond to the thickness of the silica film (*d*_*z*_(O_*t*_-O_*b*_)) and the interfacial distance (*d*_*z*_(Ru-O_*b*_)). Grey arrows in top view of (**a**) indicate the location of chemisorbed oxygen atoms underneath silicon atoms (two per unit cell). Changes in cage structures and trapping energies 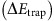 of the silica films upon Ar trapping in the cages and at the interface are shown in **d**,**e** respectively. Colour code: Si (yellow), O in the silica film (red), O chemisorbed on Ru(0001) (pink), Ru (silver) and Ar (cyan).

To explore the Ar trapping mechanism, DFT calculations were carried out for the case of silica bilayer. Silica bilayer films on *p*(2 × 1)−O/Ru(0001) (Fig. 4a, (SiO_2_)_8_/4O/Ru(0001)) with two Ar concentrations were modelled (that is, (Ar-(SiO_2_)_16_/8O/Ru(0001) in Fig. 4b with *Θ*=0.25 and Ar-(SiO_2_)_8_/4O/Ru(0001) in Fig. 4c with *Θ*=0.50). The structural changes of the silica film upon Ar trapping are quantified by following three different parameters: the changes in the thickness of the silica film (*d*_*z*_(O_*t*_−O_*b*_)) defined by the distance between O atoms in the top and bottom layers of the silica film; the interlayer distance (*d*_*z*_(Ru−O_*b*_)) defined by the distance between the top layer of Ru(0001) and the bottom layer of O_*b*_ atoms in the silica film; and the size of the cages characterized by the distance between atoms at opposite sides of the six-membered rings within the five different atomic planes in the silica film: that is, the average nearest neighbour Si–Si distance in the top layer (*d*(Si_*t*_−Si_*t*_)) and bottom layer (*d*(Si_*b*_−Si_*b*_)), the average O–O distance in the top layer (*d*(O_*t*_−O_*t*_)), the middle layer (*d*(O_*m*_−O_*m*_)) and the bottom layer (*d*(O_*b*_−O_*b*_)). Trapped Ar atoms in the nano-cages of the silica film (Ar_cage_) cause expansions at the O_*m*_ layer, where *d*(O_*m*_−O_*m*_) increased by 0.08 Å at 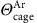
=0.25 and 0.05 Å at 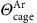
=0.50, as shown in Fig. 4d,e. The nano-cage expansion does not significantly affect the phonon vibration mode at 1,296 cm^−1^, as direction of the expansion is orthogonal to the Si–O–Si linkage perpendicular to the surface plane, which is responsible for the aforementioned vibrational mode. On the other hand, trapped Ar atoms at the interface (Ar_inter_) push the silica film away from the Ru substrate, where the inter-space *d*_*z*_(Ru-O_*b*_) increased by 0.41 Å at 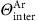
=0.25 (Fig. 4b) and by 0.88 Å at 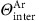
=0.50 (Fig. 4c) as compared to the interface without Ar trapping (Fig. 4a).

In order to determine the preferred trapping site, trapping energies 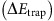 are calculated for *Θ*=0.25 and 0.50. We define


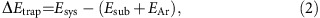


where *E*_sys_, *E*_sub_ and *E*_Ar_ are the total energies for Ar-SiO_2_/O/Ru(0001), SiO_2_/O/Ru(0001) and Ar, respectively. All energies refer to the optimized structures of the systems and subsystems. The calculated Δ*E*_trap_ (cage) is −34 meV at 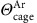
=0.25 and −29 meV at 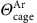
=0.50, while Δ*E*_trap_ (inter) is 331 meV at 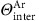
=0.25 and 176 meV at 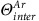
=0.50. This qualitative difference between 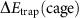
(exothermic) and 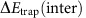 (endothermic) suggests that the cages in the silica bilayer are the thermodynamically favored Ar trapping sites over the interface, in agreement with XPS results that showed a significant larger population of Ar atoms inside the cages and earlier desorption of Ar atoms from the interface.

Moreover, Δ*E*_trap_ (cage) and Δ*E*_trap_ (inter) exhibit opposite trends with respect to *Θ*. Δ*E*_trap_ (cage) at 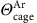
=0.50 is 5 meV higher than that at 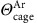
=0.25, because at higher 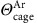, trapped Ar atoms create more stress in the silica film. As 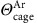 decreases from 0.50 to 0.25, *d*(O_*m*_–O_*m*_) increases from 6.28 to 6.31 Å, resulting in a release of the stress caused by Ar trapping. In contrast, Δ*E*_trap_ (inter) at 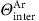
=0.50 is 155 meV lower than that at 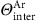
=0.25, indicating a preference at 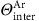
=0.50. This trend results from the competing interactions between Ar_inter_−silica/O/Ru(0001) and silica−O/Ru(0001) in the surface normal direction, which is discussed in more details by the analysis using interaction energies 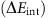, as described in Supplementary Note 1.

**Figure 5:**
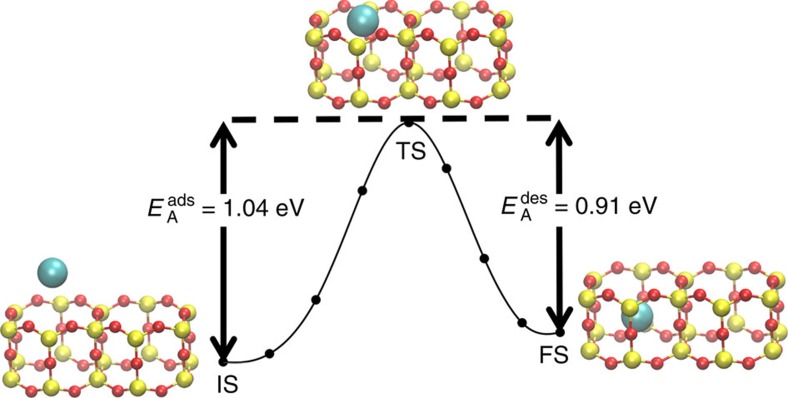
Calculated activation energies for Ar adsorption and desorption. The minimum energy path for Ar trapping from climbing image nudged elastic band calculations when all of the Ar atoms are being trapped simultaneously at 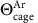
=0.25. Colour code: Si (yellow), O (red), Ar (cyan).

The activation energy for Ar adsorption (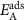
=1.04 eV) and desorption (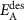
=0.91 eV) at 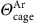
=0.25 were calculated from DFT using the climbing image nudged elastic band (CI-NEB) method^31^, suggesting that a neutral Ar atom can enter or escape from the nano-cage with an activation barrier of about 1 eV. However, as mentioned above, Ar atoms need either higher pressures or the aid from X-rays, presumably ionizing the Ar atoms, to enter the cage. Corresponding initial state (IS), transitions state (TS) and final state (FS) of the Ar trapping pathway are shown in Fig. 5. At the TS, cages in the silica film expand to allow Ar to enter; in the top layer of the silica film, *d*(Si_*t*_−Si_*t*_) increases from 6.23 to 6.26 Å, and *d*(O_*t*_–O_*t*_) increases from 5.39 to 5.55 Å. The calculated 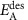 is in reasonable agreement with the estimated activation energy barrier of 1.08±0.18 eV of the aluminosilicate film from the time dependent XPS study. 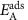 and 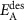 slightly increase by 0.01 eV at 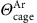
=0.50 (Supplementary Fig. 6). The high activation energy for Ar desorption results from the extreme spatial confinement effect on the trapped atoms sitting in the nano-cages, which provides a perfect benchmark platform for electronic structure studies.

While there is good agreement between the calculations and the desorption studies, it should be emphasized that further studies are needed on the adsorption process, to assess the influence of X-rays. However, note that even if Ar enters the cage as an ion, it would immediately neutralize by electron transfer from Ru(0001), which is within tunnelling range, given the ∼10 eV energy gain resulting from the difference between the first ionization energy of Ar (15.76 eV) and the O-Ru(0001) work function (∼6 eV)^28^. This is consistent with our DFT projected density of states (PDOS) analysis, showing that Ar 3p states are deep below the Fermi level.

### Tuning the framework permeability

**Figure 6:**
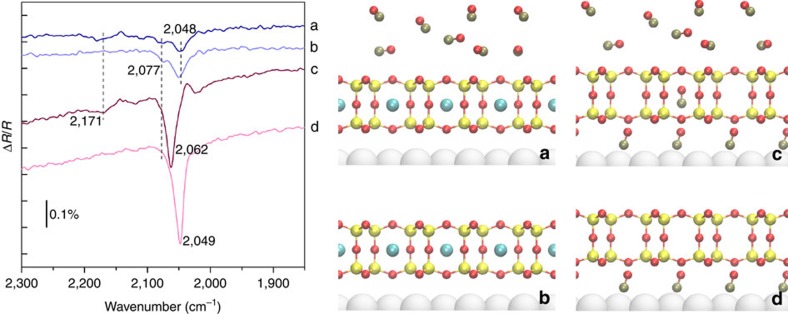
Permeability of CO through the cages. IRRA spectra and schematic representation of (**a**) O-poor Silica/Ru(0001) with trapped Ar under 3 × 10^−3^ mbar CO at room temperature and (**b**) after pumping down the CO. IRRA spectra of (**c**) O-poor Silica/Ru(0001) under 3 × 10^−3^ mbar CO at room temperature and (**d**) after pumping down the CO. The O-poor Silica/Ru(0001) is prepared by annealing the freshly synthesized silica bilayer to 1,100 K.

While previous work showed that the silica bilayer is permeable to small molecules, such as H_2_, O_2_ and CO^19^,^20^,^21^, the incorporation of Ar atoms could allow tuning this permeability in a reversible manner by restricting the passage of small molecules through the nano-cages. To explore this, we have used IRRAS to study how Ar in the cages affects the permeation of CO and subsequent adsorption on Ru(0001). Note that IRRAS is also the only reliable technique that can determine univocally the presence of the bilayer structure, as evident by the characteristic phonon vibrations at ∼1296 and ∼692 cm^−1^ (Fig. 1e and Supplementary Fig. 7)^25^. Previous experiments showed that CO molecules can pass through the framework and adsorb on the Ru(0001) surface^19^. Subsequent DFT calculations showed that an energy barrier of 0.5 eV allows the passage of CO through the six-membered rings^25^. Since chemisorbed oxygen on Ru(0001) also affects CO adsorption (Supplementary Fig. 8), the as-prepared silica bilayer was annealed in UHV at 1,100 K (that is, to make O-poor silica/Ru). This O-poor silica/Ru(0001) was then sequentially exposed to elevated pressures of Ar and CO. The IRRA spectrum in Fig. 6a shows three weak peaks for the Ar-containing silica under 3 × 10^−3^ mbar CO at 300 K. A very weak mode evident at 2,171 cm^−1^ disappears once CO is evacuated (Fig. 6b), which can be assigned to CO interacting with silanol groups from surface defects, in agreement with previous work on chabazite by Bordiga *et al*.^32^. The stronger peak at 2,048 cm^−1^ is assigned to chemisorbed CO on Ru^19^,^21^ and the small peak at 2,077 cm^−1^ may be tentatively assigned to a small population of CO in the empty nano-cages. In comparison, as shown in Fig. 6c, the silica bilayer without trapped Ar has a much stronger peak at 2,062 cm^−1^ under 3 × 10^−3^ mbar CO at room temperature, corresponding to the stretching vibration of CO with 2/3 monolayer coverage on the Ru(0001) below the silica bilayer^21^,^33^. Interestingly, this mode shifts to 2,049 cm^−1^ once CO is evacuated (Fig. 6d), indicating that some CO desorbs from Ru and the CO coverage decreases to ∼0.5 ml  (Supplementary Fig. 9)^19^. It is clear from these results that the presence of Ar in the cages substantially reduces the coverage of CO molecules that adsorb onto the Ru(0001) surface.

## Discussion

We report the room-temperature trapping of individual Ar atoms within hexagonal prism (alumino)silicate nano-cages forming a two-dimensional framework on a flat Ru(0001) surface. The trapping is confirmed *in situ* by X-ray photoelectron spectroscopy at modest pressures of only 0.5 mbar (in the presence of X-rays) to trap the Ar atoms. These trapped Ar atoms are removed from the cages upon heating to 450 K. Note that higher pressures, above 2 mbar, are needed in IRRAS experiments, showing that the presence of X-rays in XPS experiments aid in trapping the Ar atoms. The desorption activation energy was determined to be ∼1.08±0.18 eV from time-dependent XPS, in agreement with DFT calculations carried out as part of this work. While noble gases have been trapped before in three-dimensional porous materials, this is, to the best of our knowledge, the first report of a noble gas trapped in cages on a two-dimensional porous material. This is of major importance for fundamental studies as it allows the use of surface science techniques for the study of individual inert atoms in confinement, opening exciting opportunities in the field.

In addition to the possibility of using these cages for trapping small atoms and molecules and study them in confinement, the presence of Ar atoms within the cages affects the passage of small molecules through the 0.5 nm thick two-dimensional framework, allowing the reversible tuning of the permeability of the smallest molecular sieve ever reported.

## Methods

### Material synthesis

The Ru(0001) single-crystal surface was cleaned with cycles of Ar^+^ sputtering and annealing at 1,400 K. It was then exposed to 3 × 10^−6^ mabr O_2_ at 1,200 K in order to form a chemisorbed 3O-(2 × 2)-Ru(0001) overlayer. The silica and aluminosilicate bilayers were grown on the oxygen pre-covered ruthenium surface. Briefly, Si (and Al) was thermally evaporated onto the 3O-(2 × 2)-Ru(0001) surface at room temperature under 2 × 10^−7^ mbar of O_2_, followed by oxidation at 1,200 K in 3 × 10^−6^ mbar O_2_ for 10 min and slowly cooled down in O_2_ environment.

### Characterization

AP-XPS measurements were carried out at the beamline X1A1 of the National Synchrotron Light Source (NSLS) and the Coherent Soft X-ray Scattering and Spectroscopy beamline (CSX-2) of the National Synchrotron Light Source II (NSLS-II). The main chamber (base pressure 2 × 10^−9^ mbar) of the end-station was equipped with a differently pumped hemispherical analyser (Specs Phoibos 150 NAP), which was offset by 70° from the incident synchrotron light. Unless otherwise stated, the sample surface normal is 20° off the axis of the electron analyser. The Ar gas was introduced into the main chamber through precision variable leak valves for the trapping studies. Note that the spectra in Fig. 3 has broader peaks given the lower resolution at beamline X1A1 of NSLS where these were taken, as opposed to the rest of the XPS spectra, which were taken at beamline CSX-2 of NSLS-II.

The IRRAS measurements were performed in a separate UHV system (base pressure 5 × 10^−10^ mbar). IRRA spectra were collected at 4 cm^−1^ resolution using a grazing angle of 85° to the surface normal. High purity CO and Ar exposure was carried out in a separate cell sealed from the UHV chamber by a Viton O-ring.

### Computational methods

DFT calculations were performed using plane-wave basis set and the projector augmented wave formalism implemented in the Vienna Ab initio simulation package (VASP)^34^,^35^. On the basis benchmark calculations on constituents of the system (bulk ruthenium and silica)^28^, the consistent exchange van der Waals density functional (vdW-DF-cx)^36^,^37^ was used to describe the non-local vdW interactions in the Ar-silica/O/Ru(0001) system. The Ru(0001) substrate was modelled by a five-layer Ru slab. The silica/O/Ru(0001) heterojunction was modelled as bilayer silica films (SiO_2_) physisorbed on O/Ru(0001) with the unit cell size defined by *a*=5.392 Å and *b*=9.339 Å. Si atoms in the bilayer were chosen to sit on hollow sites of Ru(0001). The number of chemisorbed oxygen atoms on Ru(0001) corresponds to the coverage of *p*(2 × 1)-O/Ru(0001)^38^, which is similar to the experimentally estimated oxygen coverage of ∼0.5 ml. In Ar-(SiO_2_)_8_/4O/Ru(0001), the unit cell was used with 50% of the nano-cages of silica films or the corresponding inter-space filled with Ar atoms (*Θ*=0.50). In Ar-(SiO_2_)_16_/8O/Ru(0001), a 2 × 1 supercell was used with a 25% filling (*Θ*=0.25). In the surface normal direction, *c*=27 Å was chosen to ensure the vacuum region to be at least 10 Å thick, except for nudged elastic band calculation where the minimum of vacuum region was 7.5 Å in the initial state.

A kinetic energy cutoff of 800 eV was used to meet the required numerical convergence for vdW-DF-cx with hard pseudopotentials^39^. K-point grids of 8 × 4 × 1 and 4 × 4 × 1 were used to sample the Brillouin zone of Ar-(SiO_2_)_8_/4O/Ru(0001) and Ar-(SiO_2_)_16_/8O/Ru(0001), respectively. Ar atoms, silica films and top two layers of the Ru substrate were allowed to relax during the structure optimization until forces were smaller than 0.01 eV Å^−1^. The dipole correction method^40^ was used due to the existence of sizable surface and interface dipole moments^28^. The core-level binding energies (*E*_BE_) were calculated using the transition state model^41^. The results are extrapolated to the infinite supercell size limit, as described in our previous work^28^. All *E*_BE_ values of Ar 2p were given relative to that of Ar 2p at the interface.

### Data availability

All data that support the findings of this study are available from the authors on request.

## Additional information

**How to cite this article:** Zhong, J.-Q. *et al*. Immobilization of single argon atoms in nano-cages of two-dimensional zeolite model systems. *Nat. Commun.* **8,** 16118 doi: 10.1038/ncomms16118 (2017).

**Publisher’s note**: Springer Nature remains neutral with regard to jurisdictional claims in published maps and institutional affiliations.

## Supplementary Material

Supplementary Information
