# Supplementary material for: Immobilization of single argon atoms in nano-cages of two-dimensional zeolite model systems
Source: Nat Commun. 2017 Jul 17;8:16118. doi: 10.1038/ncomms16118 (PMC5520055; doi:10.1038/ncomms16118)
Supplement: Supplementary Information [file ncomms16118-s1.pdf]

Title of file for HTML: Supplementary Information
Description: Supplementary Figures, Supplementary Tables Supplementary Note

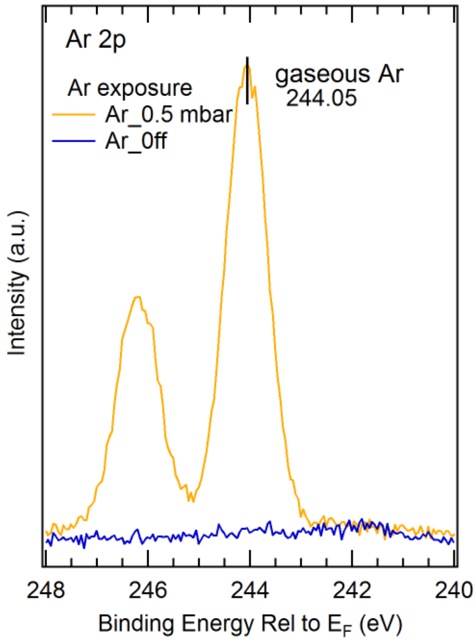

Supplementary Figure 1 | Reference experiment of Ar trapping on clean Ru(0001). AP-XPS core level spectra of Ar 2p on the Ru(0001) under 0.5 mbar Ar. The blue spectrum is obtained in UHV after 10 minutes 0.5 mbar Ar exposure. (Photon energy, $h\nu$ = 1000 eV; photoelectron emission angle, θ = 20°)

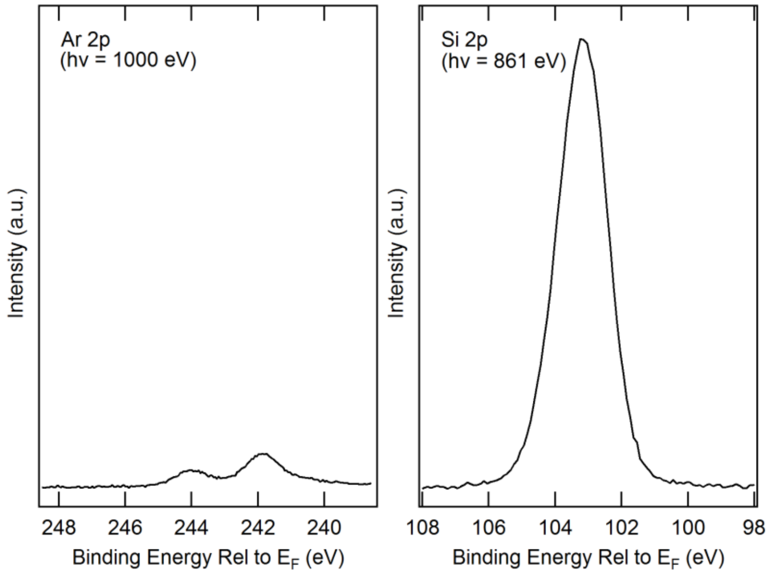

Supplementary Figure 2 | The coverage of trapped Ar. UHV XPS core level spectra of Ar 2p and Si 2p from the bilayer silica after 0.5 mbar Ar exposure. Different photon energies are used in order to have the same photoelectron kinetic energy and thus the same electron transmission function of the Specs Phoibos 150 NAP lens system (E_k ~ 759 eV). (photoelectron emission angle, θ = 20°) The number of photoelectrons detected per second from an orbital of constituent atoms can be quantified using the following equation (Handbook of X-ray Photoelectron Spectroscopy, Physical Electronics Division, Perkin-Elmer Corp., 1992),

$$I = nf\sigma\theta y\lambda AT,$$

where I is the number of photoelectrons detected per second from an orbital of constituent atoms; n is the number of atoms per cm^3 of the element of interest; f is the flux of X-ray photons impinging on the sample, in photons per $cm^{-2}s^{-1}$; σ is the photoelectric cross-section for the particular transition in cm^2 per atom; θ is the angular efficiency factor for the instrumental arrangement (angle between photon path and emitted photoelectron that is detected); y is the efficiency of production in the photoelectric process to give photoelectrons of normal energy (with final ionic state the ground state); λ is the mean free path of the photoelectrons in the sample. A is the area of the sample from which photoelectrons can be detected; T is the efficiency of detection of the photoelectrons emerging from the sample. In our experiments, these parameters (θ, y, λ, A, T) almost have the same values, and the peak intensity used in our calculation was already normalized by the photon flux [i.e., $(\boldsymbol{I_1}/\boldsymbol{I_2}) = (I_1/I_2) * (f_2)/(f_1)$]. Therefore, by comparing the ratio of peak area ($\boldsymbol{I}$) between the Ar 2p (cross section, σ ~ 0.134) and Si 2p (cross section, σ ~ 0.062), the ratio between the number of Si atoms and Ar atoms can be obtained through the equation, $n_{Si}/n_{Ar} = \boldsymbol{I}_{Si}/\boldsymbol{I}_{Ar} \times \sigma_{Ar}/\sigma_{Si}$. The total coverage of trapped Ar atoms was estimated at Θ= 0.15 ± 0.02 per nano-cage, which may be underestimated due to the existence of vitreous structures. Here, the coverage is defined as cages filled divided by the total number of cages.

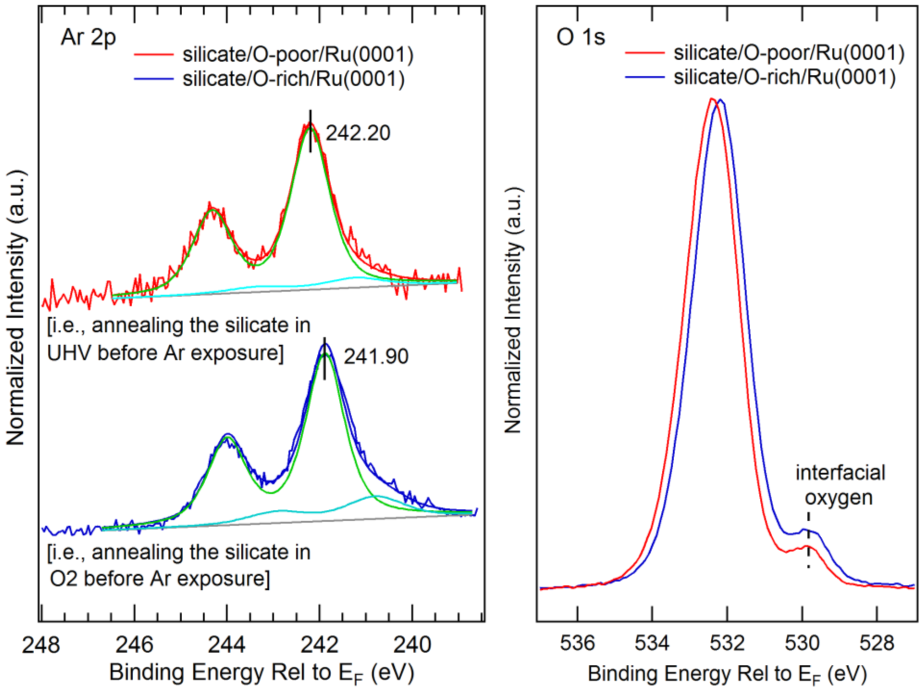

Supplementary Figure 3 | Effect of the inter-space distance on the amount of interface trapped Ar. UHV XPS core level spectra of Ar 2p and O 1s from the O-poor silicate film and O-rich silicate film. Different annealing processes before Ar exposure will change the coverage of interfacial chemisorbed oxygen on the Ru(0001) and therefore the inter-space distance between the silica film and the Ru(0001). (Photon energy, $h\nu$ = 1000 eV; photoelectron emission angle, θ = 20°)

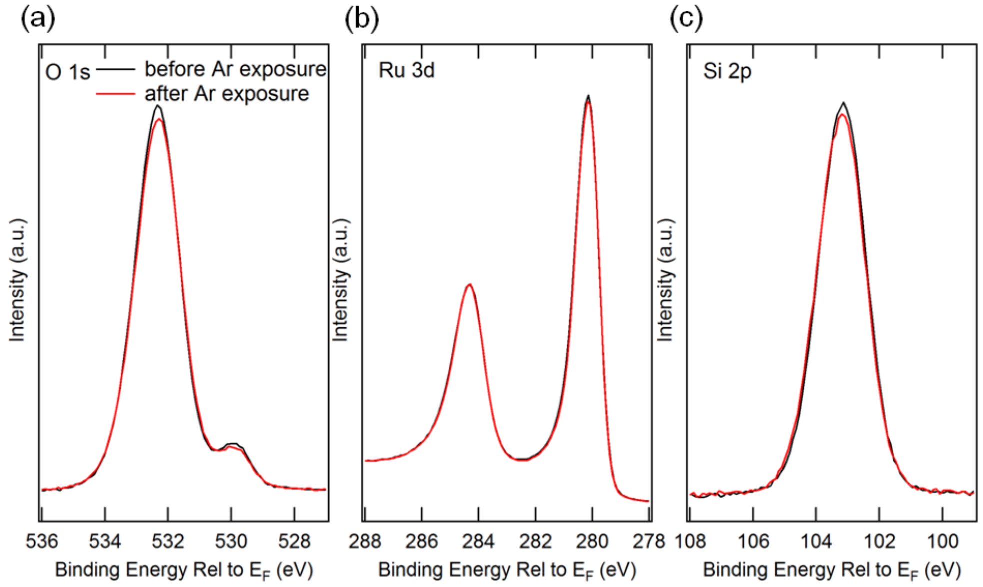

Supplementary Figure 4 | XPS of Ar-included bilayer silica film. UHV XPS core level spectra of (**a**) O 1s, (**b**) Ru 3d and (**c**) Si 2p of the bilayer silicate before and after 0.5 mbar Ar exposure. (Photon energy, $h\nu$ = 1000 eV; photoelectron emission angle, θ = 20°)

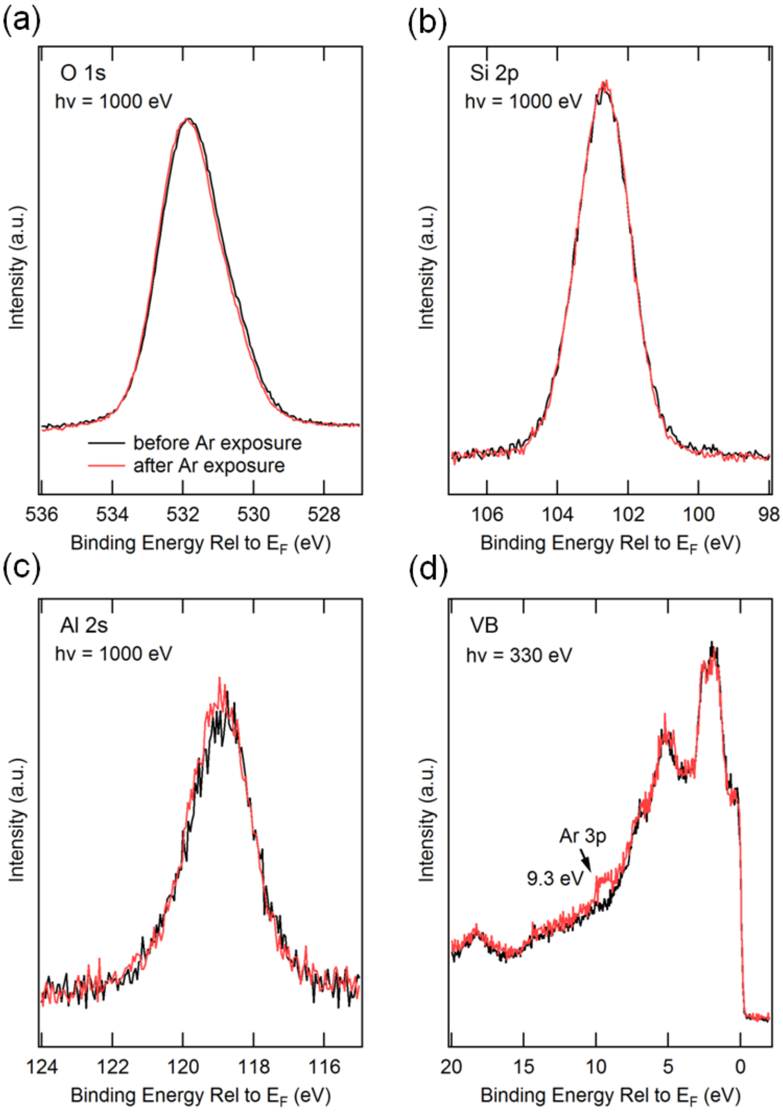

Supplementary Figure 5 | XPS of Ar-included bilayer aluminosilicate film. UHV XPS core level spectra of (**a**) O 1s, (**b**) Si 3p, (**c**) Al 2s and (**d**) valence band of the aluminosilicate ($Al_{0.16}Si_{0.84}O_2$) before and after 0.5 mbar Ar exposure. (Photoelectron emission angle, $\theta = 20°$)

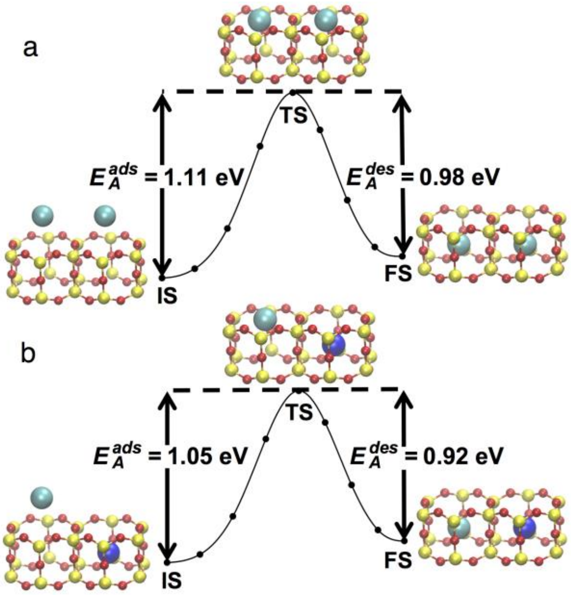

Supplementary Figure 6 | Calculated activation energies for Ar adsorption and desorption. The minimum energy path for Ar trapping from climbing image nudged elastic band calculations. (**a**) All Ar atoms are being trapped at Θ_{cage}^{Ar} = 0.50 simultaneously. (**b**) Half of the Ar atoms are fixed in the nano-cages while half of the Ar atoms are being trapped with total Θ_{cage}^{Ar} of 0.50. Color code: Si (yellow), O (red), Ar being trapped (cyan) and Ar kept fixed (blue).

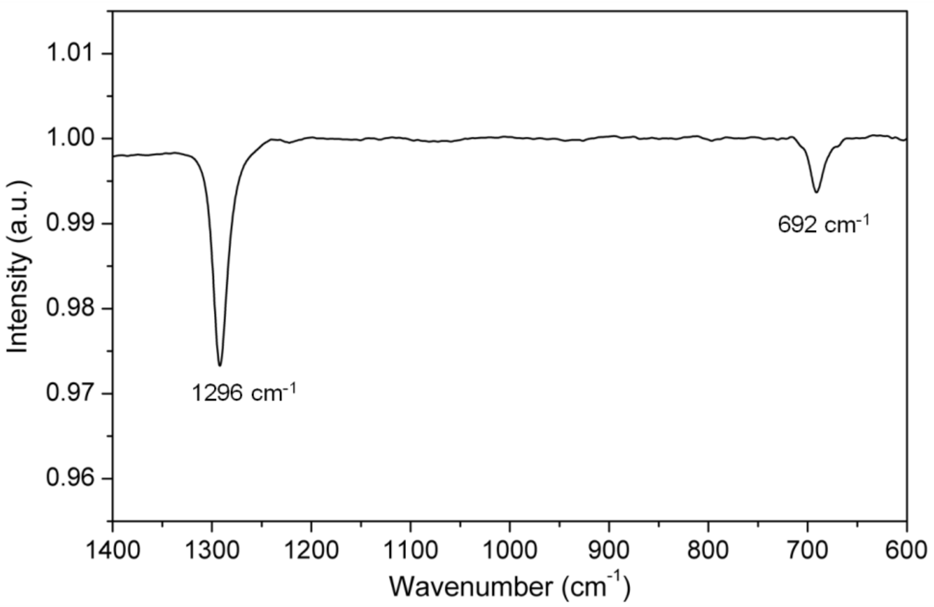

Supplementary Figure 7 | IRRAS of the as-prepared bilayer silicate film grown on Ru(0001).

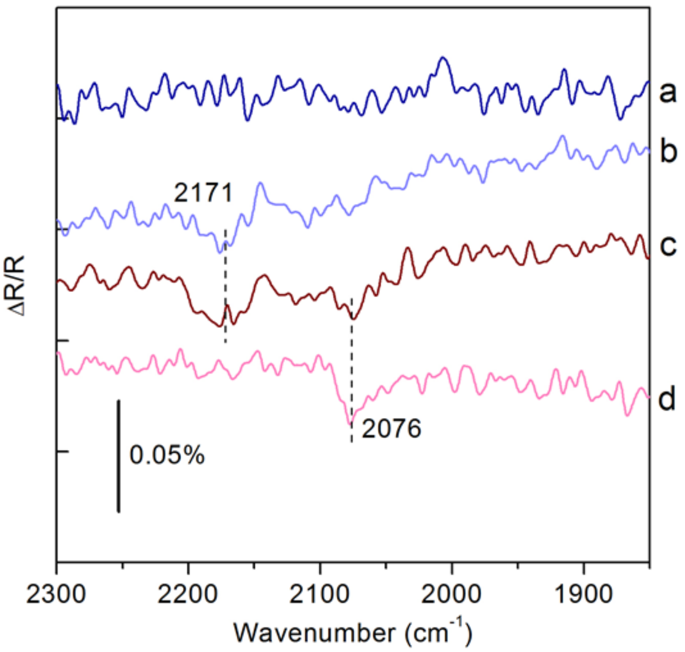

Supplementary Figure 8 | IRRAS spectra of CO adsorption on Ar trapped O-rich silica bilayer. The silica bilayer was annealed in 2x10^{-6} mbar O_2 at 1100K in order to have interfacial chemisorbed oxygen on Ru (i.e., (2x2-3O/Ru(0001)). IRRAS spectra on (**a**) Ar-containing O-rich silica/Ru in UHV, (**b**) Ar-containing O-rich silica/Ru under 3x10^{-3} mbar CO, (**c**) O-rich silica/Ru under 3x10^{-3} mbar CO at room temperature and (**d**) subsequently pumped down the CO.

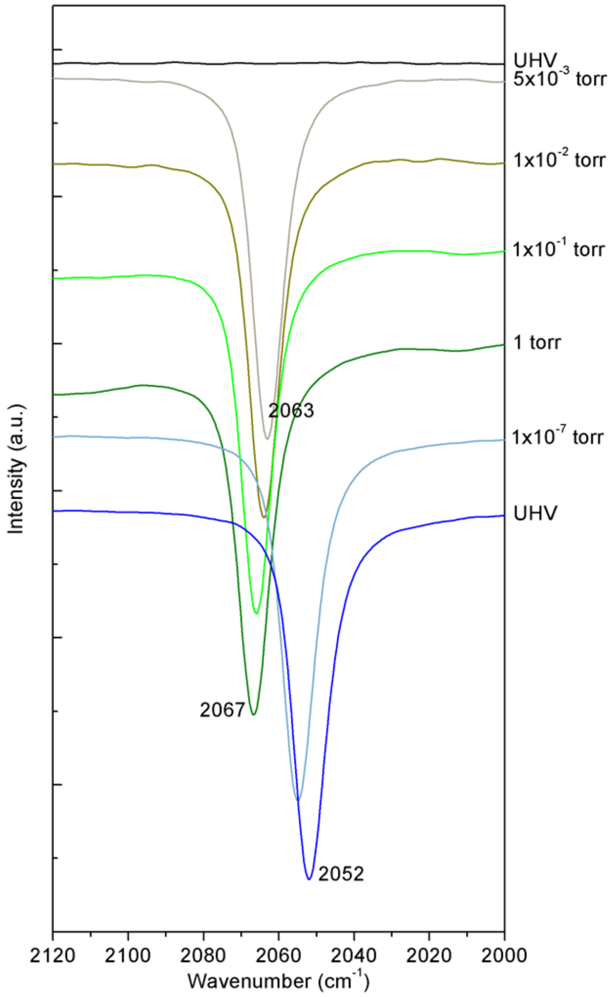

Supplementary Figure 9 | IRRA spectra of CO adsorption on clean Ru(0001).

Supplementary Table 1 | DFT calculated core level binding energies for trapped Ar atom. Simulated core level binding energy (E_{BE}) for Ar_{cage} relative to that of Ar_{inter} in a 1×1 (*a* = 5.392 Å and *b* = 9.339 Å), 2×1 (*a* = 10.784 Å and *b* = 9.339 Å) and 4×2 (*a* = 21.568 Å and *b* = 18.678 Å) supercell for Ar-$(SiO_2)_8$/4O/Ru(0001) (Θ = 0.50) and a 2×1 (*a* = 10.784 Å and *b* = 9.339 Å), 2×2 (*a* = 10.784 Å and *b* = 18.678 Å) and 4×2 (*a* = 21.568 Å and *b* = 18.678 Å) supercell for Ar-$(SiO_2)_{16}$/8O/Ru(0001)) (Θ = 0.25). All E_{BE} values are extrapolated to the infinite supercell limit. The energy unit is eV.

Supercell	1×1	2×1	4×2	Extrapolated
Θ = 0.50	2.38	1.71	1.19	1.02
Supercell	2×1	2×2	4×2	Extrapolated
Θ = 0.25	1.81	1.46	1.29	1.12

Supplementary Table 2 | DFT calculated structural changes of the silica film upon Ar trapping. Cage size (in Å) characterized as the average Si-Si distance (for atoms at opposite side of the cage) at the top (d(Si_t-Si_t)) and bottom (d(Si_b-Si_b)) layer of the silica film and the average O-O distance at the top (d(O_t-O_t)) , middle (d(O_m-O_m)) and bottom (d(O_b-O_b)) layer of the silica film. Numbers in parenthesis in Θ = 0.25 and Θ = 0.50 are the averaged distances relative to that of the structure before Ar trapping (Θ = 0).

Θ	0	0.25		0.50	
		Cage	Interface	Cage	Interface
$d(Si_t\text{-}Si_t)$	6.23	6.24 (0.01)	6.23 (0.00)	6.24 (0.01)	6.23 (0.00)
$d(Si_b\text{-}Si_b)$	6.23	6.24 (0.01)	6.24 (0.01)	6.24 (0.01)	6.23 (0.00)
$d(O_t\text{-}O_t)$	5.39	5.39 (0.00)	5.39 (0.00)	5.39 (0.00)	5.39 (0.00)
$d(O_m\text{-}O_m)$	6.23	6.31 (0.08)	6.24 (0.01)	6.28 (0.05)	6.23 (0.00)
$d(O_b\text{-}O_b)$	5.39	5.40 (0.01)	5.43 (0.04)	5.39 (0.00)	5.41 (0.02)

Supplementary Note 1 | Trends of ΔE_{trap} with respect to the concentration of trapped Ar atoms from DFT calculations. ΔE_{trap}(cage) and ΔE_{trap}(inter) exhibit opposite trends with respect to Θ, which can be understood by the analysis using interaction energy ΔE_{int}. We define

$$\Delta E_{int} = E_{sys} - (E_{sub/sys} + E_{Ar})$$

Where $E_{sub/sys}$ is the total energy of the (SiO_2)/O/Ru(0001) calculated at the optimized structure of Ar-(SiO_2)/O/Ru(0001). Inserting Ar_{inter} pushes the silica film away from the O/Ru(0001) surface. Consequently, the net ΔE_{int}(inter) results from an interplay of the loss of the interaction energy between the silica film and O/Ru(0001) and the gain in vdW energy from Ar_{inter}–silica/O/Ru(0001). At $\Theta_{inter}^{Ar} = 0.25$, the amount of Ar_{inter} is not enough to create a large enough space at the interface (d_z(Ru-O_b) = 4.25 Å) to achieve a favorable Ar_{inter}–silica/O/Ru(0001) vdW interaction. As a result, the energy penalty from separating silica–O/Ru(0001) dominates, resulting in a positive interaction energy of 187 meV. At Θ_{inter}^{Ar} = 0.50, however, as d_z(Ru-O_b) increases to 4.72 Å, the energy gain to form Ar_{inter}–silica/O/Ru(0001) overweighs the energy loss from separating the silica from O/Ru(0001), and ΔE_{int}(inter) thus becomes -26 meV. Note that this favorable case for a large amount of Ar at the interface is not reached experimentally at the low pressures used in this work, where the concentration of interfacial Ar is much smaller. It may be possible however at higher pressures.
